# Investigating the Challenges in Diagnosis and Management of Giant Cell Tumors in the Distal Phalanx: A Case Report

**DOI:** 10.7759/cureus.81180

**Published:** 2025-03-25

**Authors:** Jason S DeFrancisis, Oren D Rosenthal, Theodore C Whitford

**Affiliations:** 1 Orthopedic Surgery, Lake Erie College of Osteopathic Medicine, Bradenton, USA; 2 Medicine, Lake Erie College of Osteopathic Medicine, Bradenton, USA; 3 Anatomy, Lake Erie College of Osteopathic Medicine, Bradenton, USA; 4 Radiology, Shriners Hospital, St Louis, USA

**Keywords:** amputation, consultation, curettage, distal phalanx, giant cell reparative granuloma, giant cell tumor of bone, orthopedic hand surgery, orthopedic surgery

## Abstract

Giant cell tumors (GCTs) of bone are locally aggressive neoplasms that typically occur in the distal femur or proximal tibia. Infrequently, they may develop in the bones of the hand, including the distal phalanx. This case highlights the importance of a thorough and systematic diagnostic workup of GCTs presenting in rare and challenging locations such as the distal phalanx. A 53-year-old male presented to the clinic with a several-month history of left middle fingertip enlargement, pain, and limited mobility. Plain film X-ray and magnetic resonance imaging revealed a lesion in the left middle distal phalanx. The patient underwent curettage with a working diagnosis of giant cell reparative granuloma with focal fracture callus. Eight months later the mass recurred and amputation of the distal phalanx tip was performed. The histopathological evaluation confirmed a diagnosis of a giant cell tumor of bone. The postoperative course was unremarkable. No further treatment was required. In rare and difficult-to-diagnose tumors clinicians should consult with experienced pathologists to improve diagnostic accuracy. This approach is essential for optimizing patient outcomes, preventing treatment delays, and reducing the risk of adverse events.

## Introduction

Giant cell tumors (GCT) of the bone are characterized as locally aggressive neoplasms with a rare tendency to metastasize [1]. GCTs are uncommon, with an incidence rate of approximately one in one million people per year [2]. 80% of GCTs occur between the second and fifth decades with a higher occurrence in females [3]. The majority of cases occur in the epiphysis of long bones and occasionally extend into the metaphysis, most commonly at the knee, specifically at the distal femur or proximal tibia [4].

GCTs are locally aggressive and often present with pain, swelling, limited joint mobility, and loss of joint function [5]. Radiologic imaging modalities, including X-ray, computed tomography, and magnetic resonance imaging (MRI), play a crucial role in accurately localizing the lesion [4]. Radiographic findings commonly reveal an osteolytic lesion characterized by a well-defined, non-sclerotic margin with an eccentric location [1]. A definitive GCT diagnosis requires histopathological confirmation through biopsy [4]. Incisional biopsies are generally preferred over fine-needle aspirations due to the complexity and variability of GCTs [6]. GCTs are defined histologically by a mixture of mononuclear ovoid and spindle-shaped cells interspersed with multinucleated osteoclast-type giant cells, leading to their distinctive histopathologic profile [7]. Treatment approaches vary; however, surgical intervention is the standard, typically involving curettage [1]. In some cases, alternative measures including wide resection and amputation may be considered [4,8]. Complications of GCTs include high local recurrence rates after simple curettage, as well as the rare occurrence of lung metastases [1,4].

Although rare, GCTs have been reported to occur in less common areas of the body, including the hand, which accounts for only 2% of all GCTs [9]. Due to the rare nature of GCTs of the hand, diagnostic difficulties can arise as the lesion may share features of other giant cell-rich lesions such as a giant-cell reparative granuloma (GCRG), a benign tumor [10,11]. The following case report illustrates these challenges, highlighting the importance of accurate diagnosis and the necessity for consultation with experienced pathology specialists. This case underscores how misdiagnosis can lead to treatment delays or inappropriate intervention, potentially increasing the risk of recurrence or surgical complications.

## Case presentation

**Figure 1:**
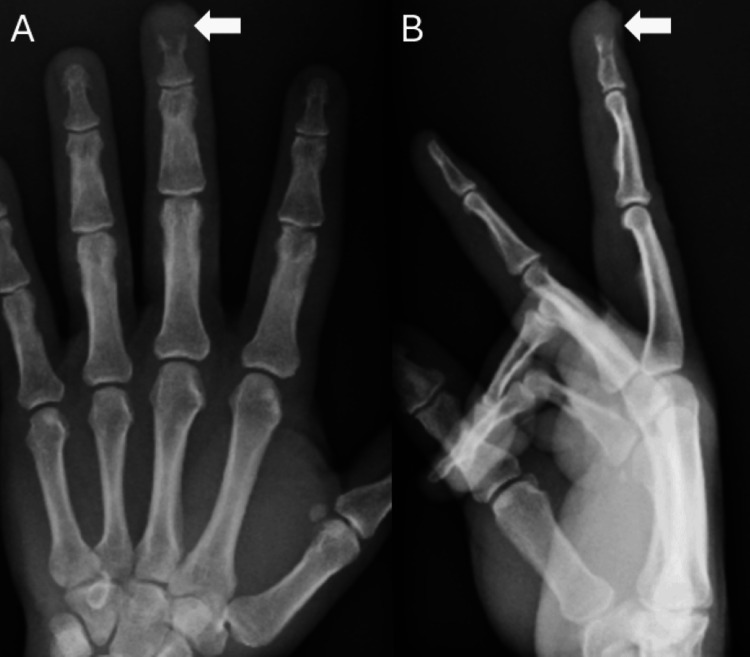
A) A posteroanterior and B) lateral radiograph of the left hand demonstrating an erosive lesion at the fingertip involving the tuft of the distal phalanx of the middle finger.

**Figure 2:**
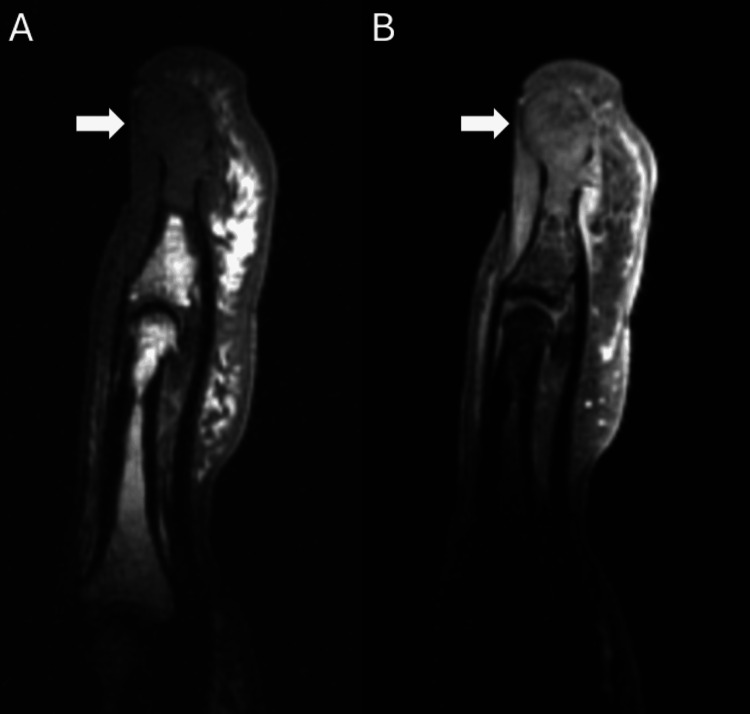
A) Sagittal T1 MRI of the left third distal phalanx, demonstrating an intraosseous mass of the distal phalanx that does not extend into the joint space. B) Sagittal T1 fat sat post-contrast MRI of the left third distal phalanx, demonstrating a solid enhancing lesion.

**Figure 3:**
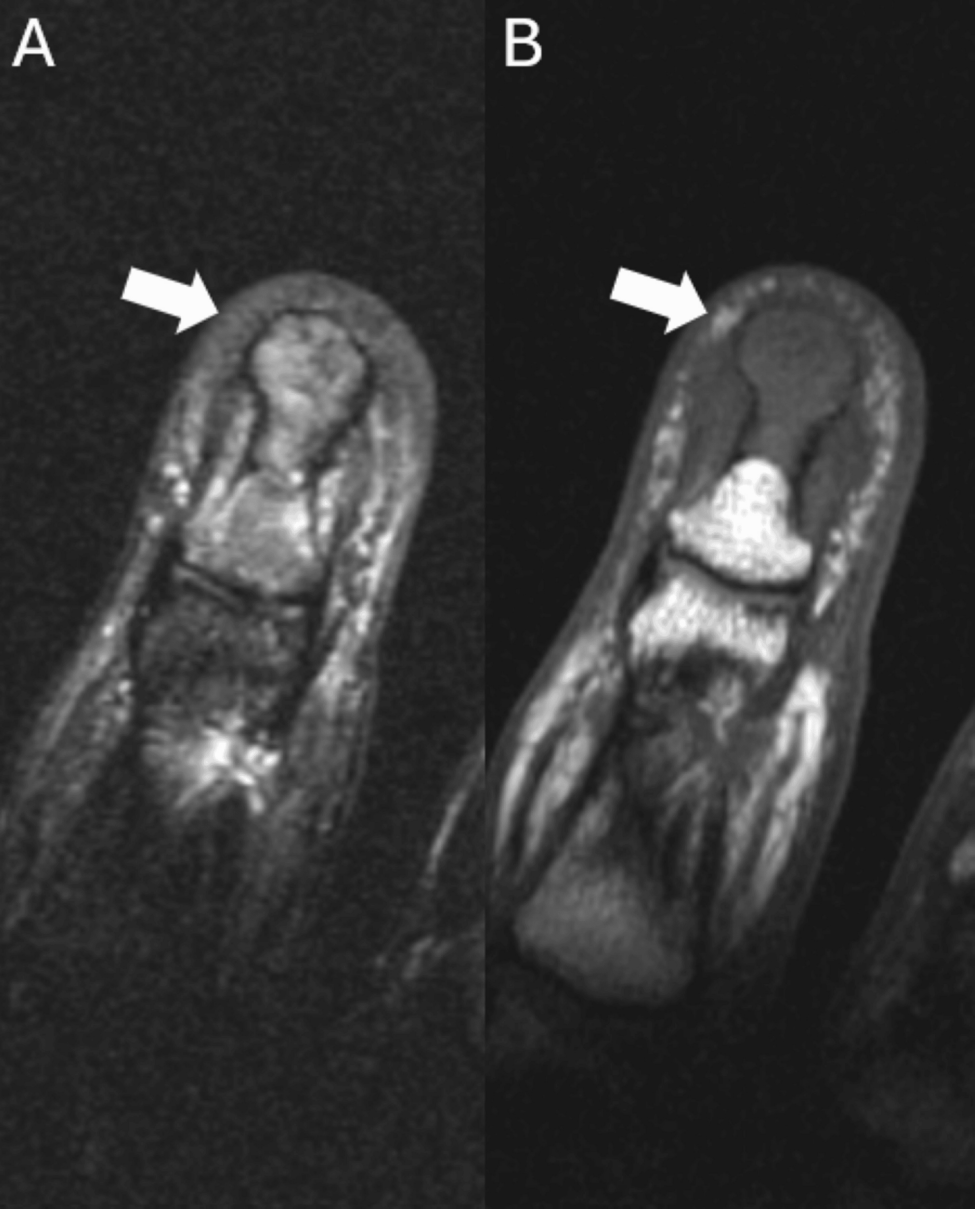
A) Coronal STIR MRI of the left third distal phalanx, demonstrating an intraosseous mass with an expansile bulbous appearance at the tuft. B) Coronal T1 MRI of the left third distal phalanx, revealing no edema within the mass and the adjacent normal bone. STIR: Short tau inversion recovery

**Figure 4:**
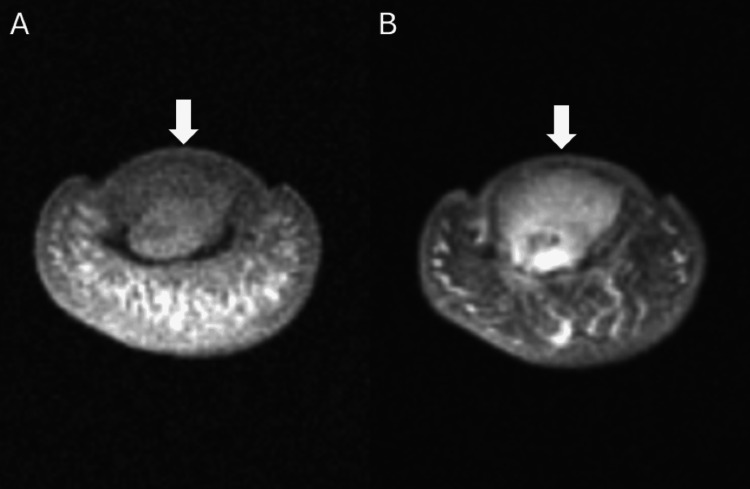
A) Axial T1 fat sat MRI of the left third distal phalanx demonstrating a solid lesion. B) Axial T1 fat sat post-contrast MRI of the left third distal phalanx of the distal phalanx, demonstrating a solid enhancing lesion.

**Figure 5:**
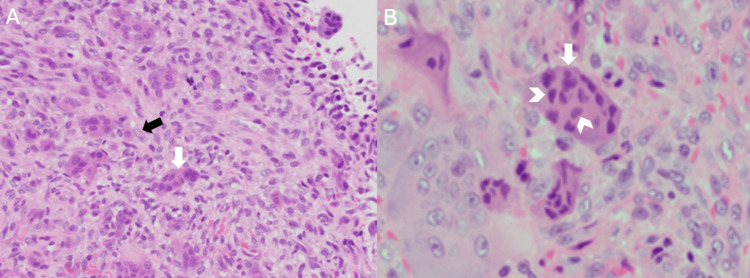
A) Micrographs of the lesion (H&E 40×), composed of an abundance of multinucleated osteoclast-like giant cells. Osteoclast-like giant cells display mitosis. The white arrow shows a multinucleated giant cell. The black arrow displays a mononuclear stromal cell. B) Zoomed-in micrograph of the lesion (H&E), displaying osteoclast-like giant cells. The white arrow shows a multinucleated giant cell. The white arrowheads point to individual nuclei within the multinucleated giant cell. (Imaging of the gross specimen was not able to be obtained but was described as consisting of multiple fragments of pink, soft tan tissue with a measurement of 1.0 × 0.7 × 0.4 cm).

A 53-year-old male patient presented to the clinic with a several-month history of enlargement of the left middle fingertip associated with considerable pain and limited mobility. Due to the mass and the patient’s ongoing symptoms, a plain radiograph of the left hand was ordered which demonstrated an erosive lesion at the fingertip (Figure 1). The lesion involved the tuft of the distal phalanx (Figure 1). A definitive diagnosis could not be established based on the plain radiograph, thus the patient underwent MRI. The successive MRI confirmed the presence of a lesion involving the distal half of the left middle phalanx and the entirety of the tuft (Figures 2-4). Differential diagnosis based on the MRI included epidermoid inclusion cyst, chronic focal infection, glomus tumor, and GCT. Due to the persistent diagnostic uncertainty a pathological evaluation was necessary for a definitive diagnosis. Despite the uncertain diagnosis, the mass was excised. Sharp dissection was utilized to expose the mass which appeared to have a fibrous lining. The mass was sharply resected and fragments were sent for cultures. The remainder of the lesion appeared pinkish-gray in color and was sent to pathology as a specimen. The remainder of the defect was thoroughly curetted until all of the pathologic-appearing tissue had been removed. Pathology reports documented an initial diagnosis of GCRG with a focal fracture callus, however, the diagnosis remained uncertain due to challenges in distinguishing the histopathologic characteristics of the lesion. Eight months after the curettage procedure the mass recurred in the same location. The patient presented with similar symptoms as the initial occurrence including swelling, pain, and limited range of motion. Following a discussion with the patient and taking into consideration the tumor’s aggressive recurrence at a relatively rapid rate, as well as the compromised function, an amputation of the distal phalanx tip, including the nail bed was performed. The procedure went well with no complications. The mass with a measurement of 1.0 × 0.7 × 0.4 cm was sent to pathology for consultation. The pathology reports documented a diagnosis of GCT (Figure 5). The patient had a recovery without complications. Follow-up displayed that the amputation provided adequate management of the lesion. The patient reported no adverse symptoms, or lesion recurrences during a follow-up phone conversation.

## Discussion

Classically, GCTs of bone grow in the distal femur or proximal tibia, however, there is potential for uncommon occurrences of GCTs in the small bones of the hand, including the distal phalanx [9]. This unusual presentation can create diagnostic difficulties as the lesion may present with clinical symptoms and features similar to those of other giant cell-rich lesions [12]. In this patient, a definitive diagnosis could not be made based on the physical exam, plain radiographs, and MRI (Figures 1-4). Lesions such as GCRGs can present with radiographic features similar to those of GCTs, particularly eccentric lytic lesions with accompanying bone remodeling [13]. The uncertainty in diagnosis prompted a pathology consultation, where the histopathological features of the lesion continued to elicit doubt surrounding the diagnosis. Ultimately a diagnosis of GCT of bone was reached after an initial diagnosis of GCRG.

GCTs and GCRGs share multiple histologic features, including osteoclastic-like multinucleated giant cells [14]. The shared features make GCTs and GCRGs difficult to distinguish from one another, however, there are histological features that help determine the diagnosis, which includes investigating the size and distribution of giant cells [15]. The giant cells in GCTs are generally found in uniform distribution among a varying number of mononucleated stromal cells [16]. GCTs also have the potential for mitotic activity and a variable number of mitotic phases can be seen [16]. In contrast, the giant cells in GCRGs are small with few nuclei and tend to be arranged in clusters around hemorrhagic zones [14]. GCRGs often develop cysts that show evidence of both recent and past hemorrhage with new osteoid formation [14]. The histopathology of the lesion in Figure 5 is characterized by an abundance of spindle and multinucleated osteoclast-like giant cells exhibiting mitosis with a significant number of nuclei. This lesion is consistent with a GCT, as the lesion displays a mixture of mononuclear ovoid and spindle-shaped cells interspersed among giant cells, which is a defining feature of GCTs [7].

Previous literature has shown that GCTs and GCRGs can be difficult to distinguish, a case report conducted by Macdonald et al. documented this difficulty in diagnosis [17]. However, the distinction in diagnosis must be made as the courses of management vary between the two pathologies [14]. Treatment of GCRGs typically involves curettage with bone grafts, which is an effective treatment for primary lesions and recurrences [11]. For GCTs, surgical intervention is the typical treatment including curettage, wide resection, and amputation [1,8]. Curettage has a higher recurrence risk compared to wide resection and amputation [1]. In hands, amputation or local resection is recommended, as curettage has been ineffective [8]. Amputation of the distal phalanx, although more aggressive, is a treatment option in which no cases of recurrence have been reported [18]. This patient’s initial procedure of curettage was inadequate as the tumor recurred, as a result, the surgical team proceeded with distal interphalangeal joint amputation as a definitive cure. Distal interphalangeal joint amputation has been associated with good functional outcomes, which was the case for this patient [19]. Following the amputation, the patient did not lose significant functional capabilities.

The significance of proper treatment emerges from the malignant potential that GCTs possess [20]. Lung metastases have been documented as a complication of GCTs [20]. Reported cases have noted their occurrence either concurrently or subsequent to the local recurrence of GCTs in the hand [20]. In addition to malignant potential, GCTs have a high recurrence rate, and GCTs of the phalanges recur more rapidly compared to other regions of the body [8]. Unlike GCTs, GCRGs have a benign course and a lower rate of recurrence, thus warranting a less aggressive management plan [14]. Although this patient did develop a local recurrence, the patient did not develop any distant metastases and has continued to stay healthy following the amputation.

## Conclusions

This case should underscore the critical importance of accurate diagnosis and timely management of GCTs in rare and challenging locations such as the distal phalanx. Clinicians must differentiate between GCTs and GCRGs, as they have different prognoses and management. In this case, diagnostic uncertainty and a delay in definitive treatment led to local recurrence, but further multidisciplinary management successfully resolved the issue. This case emphasizes the necessity for clinicians to collaborate with experienced pathology specialists in challenging cases to ensure accurate diagnosis. Such an approach not only enhances diagnostic confidence but also ensures that patients receive the most effective and evidence-based care, ultimately reducing the risk of misdiagnosis or delayed treatment, and ultimately improving patient outcomes for rare presentations.
